# Agenesis of the Right Hepatic Lobe

**DOI:** 10.1155/2012/415742

**Published:** 2012-03-21

**Authors:** Lucas Souto Nacif, Yuri dos Santos Buscariolli, Luiz Augusto Carneiro D'Albuquerque, Wellington Andraus

**Affiliations:** Transplantation Division, Gastroenterology Department, Sao Paulo University School of Medicine, Rua Dr. Enéas de Carvalho Aguiar, 255-9∘ Andar-sala, 9113 São Paulo, SP, Brazil

## Abstract

*Introduction*. Agenesis of the right lobe of the liver is a rare finding and was defined as the absence of liver tissue on the right side, with preservation of the middle hepatic vein, without previous disease or surgery. It is usually an incident finding reveled by imaging exams or during abdominal surgery. *Case Report*. A 32-year-old male patient was admitted to the hospital for abdominal discomfort and loss of appetite. Imaging studies revealed the absence of the right hepatic lobe and hypertrophied left hepatic segments. *Discussion*. Anomalies of hepatic morphology are rare and correspond to developmental defects during embryogenesis, are a rare diagnosis, and are generally diagnosed incidentally based on imaging. Agenesis or hypoplasia of the right lobe may predispose the patient to the development of portal hypertension and esophageal varices. Surgical knowledge of such anatomical agenesis is necessary for surgical planning, for the appropriate identification of intraoperative surgical findings, and for the design of the postoperative approach to therapy. *Conclusion*. Agenesis of the right hepatic lobe is a rare condition. We want to highlight the importance of understanding the condition. Surgeons must recognize the entity in order to deal appropriately with the findings.

## 1. Introduction

Agenesis of the right lobe of the liver is a rare finding [1]. It is defined as the absence of liver tissue on the right side, with preservation of the middle hepatic vein, without previous disease or surgery [2]. It is usually an incident finding revealed by ultrasonography (USG), computed tomography (CT), or magnetic resonance imaging (MR) because the condition is asymptomatic. Generally, it is associated with other anatomical alterations, such as hypertrophy of other liver segments, colonic interposition between the liver and diaphragm, right diaphragmatic hernia, portal hypertension, or an anomalously positioned gallbladder [3, 4].

## 2. Case Report

**Figure 1:**
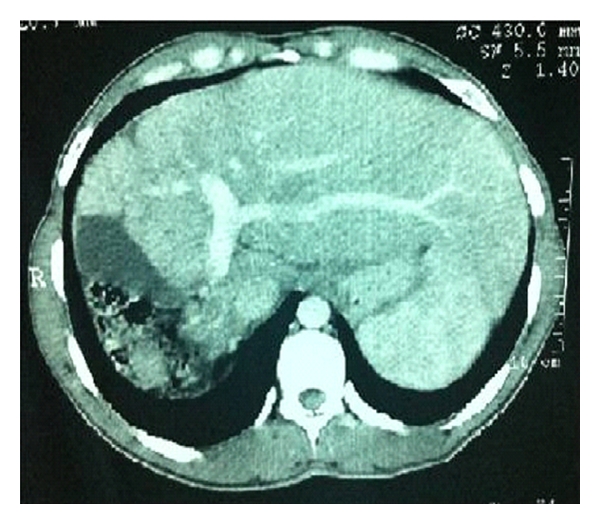
Abdominal CT: the right lobe is absent. The middle hepatic vein and extraneous segments in the left hepatic lobe are visible.

**Figure 2:**
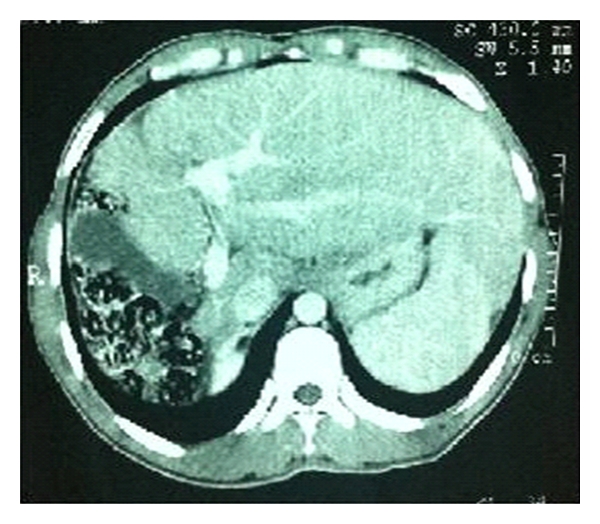
Abdominal CT: hypertrophy of the left hepatic lobe, the gallbladder is on the right of the liver in a vertical position and mild splenomegaly.

A 32-year-old male patient with insulin-dependent diabetes mellitus who was seen in an outpatient appointment with an endocrinologist was admitted to the hospital for abdominal discomfort and loss of appetite. The symptoms began few months before patient's visit. Imaging studies (Figures 1 and 2) revealed the absence of the right hepatic lobe, hypertrophied left hepatic segments, and the gallbladder placed on the right of the liver in a vertical position beside the right costal arch and the colon, and also enlargement of the splenic and portal veins and mild splenomegaly. The patient was sent to us due to these anatomical liver abnormalities, with a hypothesis of chronic liver disease. Laboratory tests and tumor markers were all normal. Endoscopy revealed only mild gastritis. Patient management after the diagnosis of agenesis of the right hepatic lobe included only observation with imaging exams and symptomatic drugs. It was suggested a mandatory medical followup with annual exams: laboratory, imaging, and endoscopy.

## 3. Discussion

Developmental anomalies of the right lobe of the liver were first reported in 1870 by Heller [4]. Anatomical variations are common and occur during normal development of the organ. They correspond to variations in the distribution of liver territories [2]. Conversely, anomalies of hepatic morphology are rare and correspond to developmental defects during embryogenesis [5]. According to Pages et al. [6], anomalies of morphology related to developmental defects can be categorized as follows: *agenesis *(absence of a lobe that is replaced by fibrous tissue); *aplasia *(one of the lobes is small and its structure is abnormal, with few hepatic trabeculae, numerous bile ducts, and abnormal blood vessels); or *hypoplasia *(one of the lobes is small but is normal in structure) [6]. According to this classification, our case would be categorized as agenesis.

 Agenesis of the right lobe of the liver is a rare diagnosis and is generally diagnosed incidentally based on imaging (USG, CT, and MR) [7]. Classically, the image reveals the absence of a right lobe with left- and caudate-lobe hypertrophy. Differential diagnoses such as postnecrotic cirrhosis, bile duct obstruction, venoocclusive disease, and hydatid disease (any of which may cause severe atrophy of the right liver) must be excluded [3].

 The recognition of this entity is facilitated by examining the right hepatic vein, right portal vein, and its associated branches if there is no right hepatic duct dilation. Evidence of these structures eliminates the possibility of a congenital liver anomaly and these findings are essential in order to establish a CT diagnosis [2, 4].

 In cases of this congenital liver anomaly, the gallbladder is often placed on the right of the liver against the diaphragm in a vertical position [2]. Malformations of the right hemidiaphragm, pulmonary alterations, and the modification of intestinal rotation may also occur [2, 3]. Agenesis or hypoplasia of the right lobe may predispose the patient to the development of portal hypertension and esophageal varices, especially when the left lobe is not enlarged [8]. In these cases, patients may present with episodes of bleeding esophageal varices before they are 30 years old. The probable etiology of portal hypertension appears to be due to a reduction in the number of intrahepatic branches of the portal vein that are not compensated for by increased density of the left lobe vasculature [8]. However, there are reports of patients with no increase in vascularization of the left hepatic lobe and no evidence of portal hypertension. Thus, the physiopathology of portal hypertension in patients with agenesis of the right lobe or hypogenesis remains to be elucidated [2].

 Surgical knowledge of such anatomical agenesis is necessary for surgical planning, for the appropriate identification of intraoperative surgical findings and for the design of the postoperative approach to therapy [1, 3]. However, knowledge of the diagnosis and adequate orientation are much more important for the patient himself because the chance that he will present with a disease related to this anatomical alteration is quite high. Gallbladder, liver, or colon problems may require biopsy or even surgery. We share the opinion with Fuertes et al. [9] that this entity is not a contraindication for laparoscopic cholecystectomy, but the previous knowledge of the condition is indeed appropriated. The gallbladder may require a different placement of laparoscopic instruments and may require a different strategy.

## 4. Conclusion

Agenesis of the right hepatic lobe is a rare condition. However, we want to highlight the importance of understanding the condition. Surgeons must recognize the entity in order to deal appropriately with the findings. The patient needs to be educated regarding the benign evolution of the condition and future implications.
